# 2-in-1 Phase Space Sampling for Calculating the Absorption Spectrum of the Hydrated Electron

**DOI:** 10.1021/acs.jctc.4c00106

**Published:** 2024-05-10

**Authors:** László Turi, Bence Baranyi, Ádám Madarász

**Affiliations:** † Institute of Chemistry, ELTE, Eötvös Loránd University, Pázmány Péter sétány 1/A, Budapest H-1117, Hungary; ‡ Research Centre for Natural Sciences, Magyar Tudósok Körútja 2, H-1117 Budapest, Hungary

## Abstract

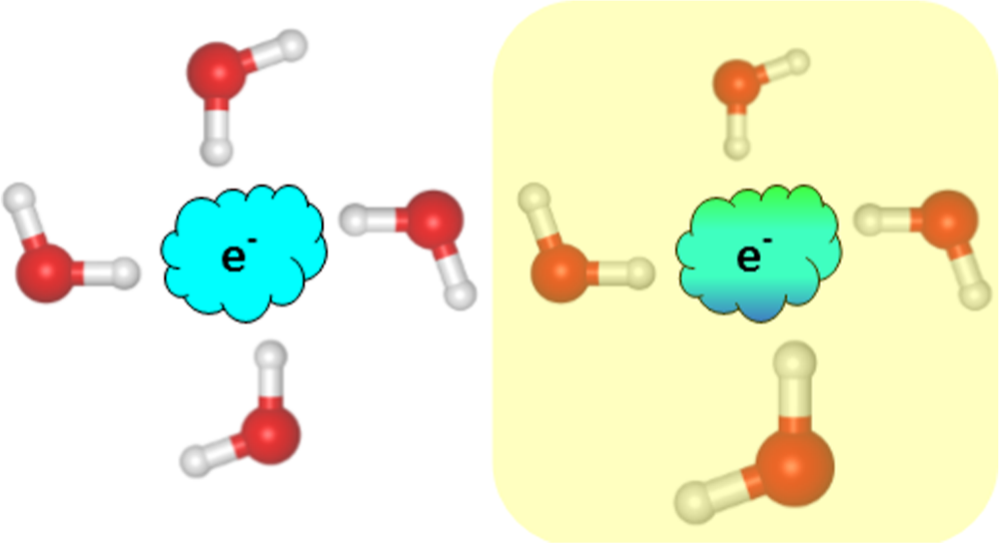

The investigation of vibrational effects on absorption spectrum calculations often employs Wigner sampling or thermal sampling. While Wigner sampling incorporates zero-point energy, it may not be suitable for flexible systems. Thermal sampling is applicable to anharmonic systems yet treats nuclei classically. The application of generalized smoothed trajectory analysis (GSTA) as a postprocessing method allows for the incorporation of nuclear quantum effects (NQEs), combining the advantages of both sampling methods. We demonstrate this approach in computing the absorption spectrum of a hydrated electron. Theoretical exploration of the hydrated electron and its embryonic forms, such as water cluster anions, poses a significant challenge due to the diffusivity of the excess electron and the continuous motion of water molecules. In many previous studies, the wave nature of atomic nuclei is often neglected, despite the substantial impact of NQEs on thermodynamic and spectroscopic properties, particularly for hydrogen atoms. In our studies, we examine these NQEs for the excess electrons in various water systems. We obtained structures from mixed classical-quantum simulations for water cluster anions and the hydrated electron by incorporating the quantum effects of atomic nuclei with the filtration of the classical trajectories. Absorption spectra were determined at different theoretical levels. Our results indicate significant NQEs, red shift, and broadening of the spectra for hydrated electron systems. This study demonstrates the applicability of GSTA to complex systems, providing insights into NQEs on energetic and structural properties.

## 1 Introduction

The hydrated electron is a curious and challenging species. Since its original discovery and identification,^1^ the hydrated electron has grasped and regrasped the scientific attention in several waves. Most likely, the reason for this recurrent interest originates from the deceiving simplicity of the system, with one excess electron being embedded in a bath of water molecules. Experimentalists and theoreticians alike very often use the hydrated electron system, in its bulk or cluster forms, to develop and test new experimental setups and/or theoretical methods. Such parallel efforts may inspire new ideas and lead to a symbiotic interplay between experiment and theory.

Numerous reviews have been dedicated to systematically collecting the most important findings on the hydrated electron topic. Of the latest such reviews, we cite the work of Young and Neumark^2^ from the experimental side, while on the theoretical side, the focus of the present work, we mention the more recent reviews by Turi and Rossky^3^ and by Herbert and Coons^4^ providing comprehensive summaries on the theory and simulations of the hydrated electron.

The most recent theoretical attempts aim at at least three more-or-less distinct directions: (a) to improve the one-electron pseudopotential in the mixed quantum-classical molecular dynamics (QCMD) approach to achieve more accurate predictions of the experimental observations, namely, the resonant Raman spectrum or the temperature dependence of the absorption spectrum;^5,6^ (b) to introduce more precise hydrated electron potential energy surfaces in the simulations using ab initio MD (AIMD) techniques^7^ or, slightly more approximately, machine learning (ML)-based methods;^8,9^ and (c) to treat the nuclei quantum-mechanically, which opens the possibility of examining nuclear quantum effects (NQEs) in hydrated electron systems.^8,10^

The present work belongs to the third category where one aims to go beyond the simple classical treatment of nuclei and investigate the effect of nuclear quantization on the properties of the hydrated electron. The history of such attempts started with the work of Neria et al., who computed the excited-state lifetime of the hydrated electron using the frozen Gaussian approximation for the nuclei.^11,12^ Prezhdo and Rossky evaluated the decoherence time scale and corrected the QCMD simulated excited-state lifetime taking decoherence into consideration.^13,14^ Later, a quantized time correlation function formula was introduced by Borgis and his co-workers^15,16^ to compute the excited-state lifetime of the hydrated electron in bulk and clusters.^17,18^ The formalism was also applied for the NQEs on the shape of the absorption spectrum of the hydrated electron.^19^ The excited-state lifetime of a  cluster was also evaluated directly using ring polymer MD simulations for the nuclei with a one-electron Hamiltonian for the electronic degrees of freedom.^20^ In all the above cases, NQEs proved to be substantial.

Recently, Lan et al. demonstrated the appearance of significant NQEs in their ML potential-based MD simulations of the bulk hydrated electron.^8^ Most notably, the electron localizes in single-cavity structures in classical MD, while twin (or double)-cavity structures also appear in 5–10% of the configurations in path-integral MD (PIMD). Interestingly, and relevant to the present paper, the absorption spectrum of the double-cavity structures is red-shifted compared to the single-cavity spectrum. In addition, large NQEs were observed in the excess-electron–hydrogen/oxygen radial distribution functions. The second peak seen in mixed QCMD simulations at around 4–5 Å^21,22^ disappeared in the e-O radial distribution function in the PIMD simulations. Subsequently Lan et al. developed a new ML potential for the bulk hydrated electron and managed to reproduce the temperature dependence of the absorption spectrum in ML potential-driven classical MD simulations.^9^ NQEs were, however, not investigated in this study. In the most recent attempt, Gijón and Hernández performed PIMD simulations on water cluster anions using one-electron pseudopotential QCMD simulations to investigate NQEs on energetic and structural properties of the clusters.^10^ They found that NQEs are small on the investigated properties, but of the two major electron-binding motifs, interior and surface states,^23,24^ the interior states become thermodynamically more favorable at smaller cluster sizes relative to the surface states than in simulations where nuclei were treated classically.

In the present paper, we decided to re-examine NQEs on the absorption spectrum of the hydrated electron system. The two most commonly used methods for accounting for the effect of molecular vibrations in the calculation of absorption spectra, thermal sampling^25^ and Wigner sampling,^26^ were recently reviewed by Nogueira and González.^27^ Thermal sampling is usually performed using MD simulations, yielding an ensemble corresponding to a classical Boltzmann distribution.^28−34^ The advantage of this method is its applicability to complex anharmonic systems, but the drawback is the absence of zero-point energy. In Wigner sampling, an ensemble is generated from an optimized structure, incorporating a zero-point energy based on normal mode vibrations. The applications of Wigner sampling successfully reproduced the broadening and shifting of peaks in several experimental absorption spectra.^35−45^ However, its limitation lies in its applicability only to rigid molecules. Attempts have been made in the literature to combine the advantages of both techniques, aiming to work for flexible systems and include zero-point energies.^46−52^

Recently, an approximative way of quantizing classical simulation results was introduced by Madarász and his co-workers as the generalized smoothed trajectory analysis (GSTA) method.^53^ It was shown that Berens et al.’s original idea^54^ about the quantum correction on thermodynamic properties can be extended to structural properties if the quantum correction is applied in the time domain instead of the frequency domain. In GSTA, one can incorporate NQEs with the filtration of the classical coordinates and velocities using a kernel function derived from the energy of the quantum harmonic oscillator. The quantized properties can be readily computed from the filtered trajectories. GSTA was successfully applied to different water phases to analyze dynamic and structural properties.^53,55^ The heat capacities of 100 organic liquids were also computed, and the results also validated the GSTA method.^56^ NQEs were investigated by the GSTA method in the UV–vis absorption spectrum of two acridine derivatives.^57^ The spectrum was computed as an average from hundreds of structures taken from AIMD simulations. Here, we concluded that GSTA works more reliably in anharmonic systems than in Wigner sampling. The main reason is that Wigner sampling uses second derivatives in a local energy minimum and generates structures from this information, while GSTA uses the trajectory. This means that GSTA uses more and more diverse information about the potential energy surface than Wigner sampling does. A brief description of GSTA can be found below in the Methods section.

In the present work, our aim is to apply GSTA for the hydrated electron systems, evaluate the appearance of NQEs on the optical absorption spectra, and, in general, provide an additional test for the applicability of the method. We note that it is much more challenging to study NQEs in systems that cannot be accurately modeled with an optimized structure such as the hydrated electron. Furthermore, the case of the hydrated electron spectrum is, by far, not a trivial one. The spectrum of the bulk hydrated electron was determined more than 40 years ago featuring a broad and highly asymmetric band with a maximum centered at 1.72 eV,^58^ but the theoretical reproduction of the spectrum has still remained a stubborn problem.

Early one-electron pseudopotential QCMD simulations were not able to quantitatively reproduce the position and the shape of the spectrum, and, in particular, significantly underestimated the high-energy part.^15,21,59−61^ Despite their inadequacy, these simulations clarified the fundamental physical aspects that determine the shape of the spectrum. Most notably, it was found that three s–p type transitions dominate the spectrum and that solvent fluctuations also significantly contribute to the spectrum’s shape and breadth.^21^ Subsequent improvements of the pseudopotential resulted in a more satisfactory agreement of the position of the spectral maximum with experiment^2,22,62^—the latest reoptimized pseudopotential was even able to capture the temperature dependence of the maximum of the absorption spectrum^6^—but the long tail has still eluded the theoretical efforts. The first real success came with the time-dependent density functional theory (TD-DFT) calculations by Herbert and his co-workers. They pointed out that a carefully crafted DFT functional (i.e., LRC-μBOP functional) in the TD-DFT scenario results in near quantitative agreement between the computed optical spectrum and experiment,^63−65^ for configurations taken from one-electron QCMD simulations. In addition to the cavity contribution to the spectrum (the dominant part in QCMD simulations), they identified a significant diffuse part and an overlap component with water molecules. The next theoretical level, calculation of the absorption spectrum of the hydrated electron based on full AIMD simulations is still plagued by severe technical limitations, like finite simulation box size effect and the level of accessible quantum chemistry used, as was demonstrated by bulk hydrated electron AIMD simulations of Park and Schwartz using the LRC-ω PBE level of theory.^7^ The most recent ML potential-based PIMD simulations that, in principle, included NQEs, as well, also failed to reproduce the spectrum satisfactorily,^8^ a fact that is expected to draw more attention in the near future.

The available data for the absorption spectra of  water cluster anions are significantly more limited than for the bulk hydrated electron. In fact, these spectra were predicted theoretically almost 10 years before their measurement.^23^ Ayotte and Johnson measured the spectra for size-selected water cluster anions containing water molecules in the *n* = 6–50 range.^66^ Bartels’ subsequent spectral moment analysis^67^ motivated a series of experimental^2,68,69^ and theoretical studies^24,70,71^ on the existence and physical properties of various water cluster anion isomers. It is understood that interior-state clusters feature absorption spectra that are similar in shape and position to the bulk spectrum, while the spectra of the surface species are significantly red-shifted and narrow, compared to the bulk.^24,70^

The goal of the present paper is to examine and test the applicability of the GSTA method for the optical spectrum of bulk and cluster hydrated electron systems. On the one hand, the optical spectrum of the hydrated electron represents a challenging scientific problem, while on the other hand, abundant experimental and simulation data are available for comparison. Furthermore, with this analysis, we hope to be able to gain a molecular-level qualitative picture of the underlying quantum effects, a huge advantage of the GSTA method. We also intend to directly compare the results of the GSTA method to another quantization scheme, namely, the correlation function technique,^17,18^ that was also applied for the hydrated electron spectrum. We believe that eventually this comparison can be further utilized to extend the applicability of the GSTA technique for quantum corrections of other physical quantities involving correlation functions.^19^

For this purpose, we perform one-electron pseudopotential QCMD simulations on the hydrated electron systems, both in bulk and two  clusters with *n* = 45 and *n* = 200 water molecules. In order to assess NQEs, we apply the GSTA^53^ for the QCMD trajectories and compute the absorption spectra along these “GSTA-quantized” trajectories. The computed quantized spectra are then compared to the classical spectra (computed directly along the nonquantized QCMD trajectories) and also with spectra computed previously using the correlation function quantization procedure of Borgis et al.^19^ For comparison to higher-level methods, we also perform TD-DFT calculations on selected QCMD configurations and analyze the resulting absorption spectra.

## 2 Methods

### 2.1 QCMD Simulations

First, we performed one-electron QCMD simulations on the water cluster anions and bulk hydrated electron employing the QCMD simulation technique developed by Rossky et al.^72^ Since we used this method on several occasions,^24,70,73−75^ and the technical details of the method are well-documented, we limit ourselves to the key features of the simulations here. In particular, the one-electron Schrödinger equation for the excess electron is solved in the field of classical water molecules modeled by a flexible simple point-charge model (SPC + flex).^76^ The interaction of the excess electron and the water molecules is represented by a pseudopotential; in the present case, we apply the Turi–Borgis (TB) pseudopotential.^22,77^ The TB potential, despite its simple analytical form, has proved to provide semiquantitative agreement on many characteristic properties of the bulk hydrated electron and water cluster anions.^18,22,24,63,64,70^ Most recently, two modifications have been proposed to the original form,^5,6^ but for the sake of consistency with our previous studies, we remain to use the original TB potential. The wave function of the excess electron is represented by its wave function on a finite grid evenly distributed in a cubic box. The time-independent Schrödinger equation for the excess electron is solved using an iterative and block Lanczos procedure. The time evolution of the water bath is driven by the combined force of the electron (quantum force) and water molecules (classical force). The quantum part of the force is dictated by the Hellman–Feynman theorem. The dynamics is adiabatic with the classical molecules moving on the ground-state excess electronic potential surface.^72^ The Verlet algorithm is used to integrate the equations of motion with a time step of 0.5 fs.^78^

Equilibrium trajectories are generated for *n* = 45 and 200 water cluster anions and for the bulk hydrated electron embedded in a bath of 1600 molecules in a cubic simulation cell using periodic boundary conditions. The size of the simulation box is 36.34 Å for the bulk and 363.4 Å for the clusters. The length of the trajectories is 200 ps. The evaluation of all the interactions for the bulk simulation is performed using a spherical cutoff smoothed by a tapering function.^24,70,73−75^ The neglect of the long-range interactions beyond this range has no such effect on the hydrated electron properties that would influence the conclusions of the present paper. The wave functions are evaluated on a cubic grid of 18.17 Å length using 16 × 16 × 16 discrete grid points during the dynamics. The simulations were carried out in the microcanonical ensemble and were started from previously equilibrated and thermostated trajectories. The equilibrated temperature of the clusters (consistent with the internal kinetic energies) is set to 200 K, a typical cluster simulation temperature used to model warmer cluster conditions in experiments.^24,70,73−75^ The temperature of the bulk is chosen to be 300 K, the temperature of the experimental spectrum.^58^

The frequency-resolved absorption spectrum is given by the well-known Kubo formula^79^
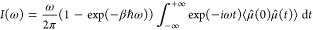
1where ω stands for the frequency and  denotes the time-dependent electronic dipole moment operator. In the so-called slow-modulation limit for a given set of atomic coordinates **x** = (*x*_1_, *y*_1_, *z*_1_, *x*_2_, *y*_2_, *z*_2_,..., *x*_*N*_atoms__, *y*_*N*_atoms__, *z*_*N*_atoms__), one obtains the electronic ground-state absorption spectrum
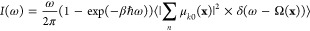
2

The transition frequencies Ω(**x**) and the corresponding transition dipole moments μ_*k*0_(**x**) depend on the atomic coordinates. The spectral calculations are performed on 1996 configurations in each investigated case. The wave functions are evaluated on a cubic grid of 36.34 Å length using 32 × 32 × 32 discrete grid points in the spectral calculations. This procedure results in the classical spectrum.

### 2.2 Quantization with GSTA

In the present study, we apply the GSTA method^53^ to include NQEs in the spectral calculations. In the GSTA method, the classical nuclear trajectories are convoluted with an appropriate kernel function corresponding to the harmonic oscillator approach to obtain the quantum-corrected atomic coordinates
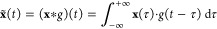
3where **x**(*t*) denotes the coordinates of the classical trajectory, while  stands for the set of filtered (quantized) atomic coordinates. Convolution is represented by an ∗. The filtering function *g* is defined as
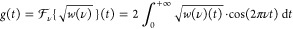
4where  indicates Fourier transformation in the frequency domain ν and *w*(ν) is the weighting function which gives the ratio of the energies of the quantum and classical harmonic oscillators
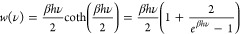
5where coth is the hyperbolic cotangent function, , *k*_B_ is the Boltzmann constant, *T* is the temperature, and *h* is the Planck constant. In the present case, the convolution is done by scanning the trajectories with a moving window of 100–200 fs. The program code used for filtration is available on GitHub.^80^

Qualitatively, GSTA works as a weighted moving average of the coordinates. In the time domain, the *g* function gives the weights. As a result of the filtering, low-frequency movements practically do not change, while the amplitude of higher-frequency vibrations is significantly amplified according to the *w* weight function in the frequency domain. At very high temperature, the *w* weight function approaches the uniform distribution according to the classical equipartition theorem. Then the *g* function approaches the Dirac delta function in the time domain, which means that filtration does not change the trajectory. This illustrates that at sufficiently high temperature, the system behaves classically.

Here, first, the GSTA quantization procedure is applied for the classical trajectory resulting in the quantized trajectory. For the calculations of the absorption spectra, configurations in every 100th frame from both the classical and filtered (quantized) trajectories were used. All in all, we use 1996 classical configurations and the corresponding 1996 “quantized” configurations for the spectral calculations. Analysis (including the calculation of the optical spectra) is performed on both the classical and quantized configurations. In particular, the quantized spectra were calculated with the application of eq 2 using the set of quantized atomic coordinates . The results are then compared.

We note here that previously, the quantized spectrum of the hydrated electron has been obtained with the application of the quantum harmonic correction scheme on the dipole moment autocorrelation function in eq 1.^19^ For this procedure, however, it is necessary to calculate the autocorrelation functions (ACFs) of the energy gap and the transition dipole moment vectors.

### 2.3 TD-DFT Calculations

To provide more insights into our analysis and further support our QCMD-based conclusions, we also performed TD-DFT calculations on water cluster anion configurations selected from both the classical and the quantized trajectories. Here, we considered only the larger *n* = 200 cluster size for TD-DFT calculations due to its compact, interior excess electronic state localizing in a solvent cavity.^23,24,71^ Since this structure is analogous to that of the bulk hydrated electron, direct comparison with the QCMD-based calculations for both the large cluster case and the bulk is feasible. On the other hand, the *n* = 45 hydrated electron clusters do not bind the electron in cavities but rather the excess electron is delocalized in diffuse states on the surface of the cluster, the electron density extending far outside the nuclear framework.^23,24,71^ Reliable representation of such states is difficult and would require an extremely large and diffuse basis set. For this reason, we decided to focus only on the *n* = 200 clusters.

Based on these general considerations, we used the SCAN-D3(BJ) functional^81^ in the TD-DFT calculations which performs remarkably well among several meta-GGA and even some hybrid functionals.^82^ We applied the relatively modest ma-svp basis set^83^ augmented by a set of diffuse s-functions on the hydrogen atoms with a coefficient of 0.02974. Here, we repeat our argument that interior states of the excess electron in large hydrated electron clusters are relatively compact; therefore, modest basis sets are expected to capture the most important electronic properties of the excess electron. Since the same TD-DFT method (functional and basis set) is applied for the classical and the quantized configurations, we also expect that inaccuracies in the functional and the basis set would not alter our conclusions on the effect of the quantization procedure with respect to the classically computed quantities. All in all, we chose a relatively cheap, nevertheless potentially reliable method to compute the absorption spectra of the *n* = 200 cluster anion. For the TD-DFT calculations, we used the ORCA program package.^84,85^

## 3 Results and Discussion

### 3.1 Absorption Spectra

First, we calculated the optical absorption spectra of (a) the *n* = 45 water cluster anion with the surface excess electronic state, (b) the *n* = 200 cluster where the electron is localized in the interior of the cluster, and (c) the bulk hydrated electron. All spectral calculations are carried out on equilibrium (classical) configurations taken out from mixed QCMD simulations (classical spectra) and on the quantized configurations we received after performing the GSTA quantization procedure on the classical trajectories (quantized spectra). The spectra contain transitions from the ground state to the first 11 excited states.

**Figure 1:**
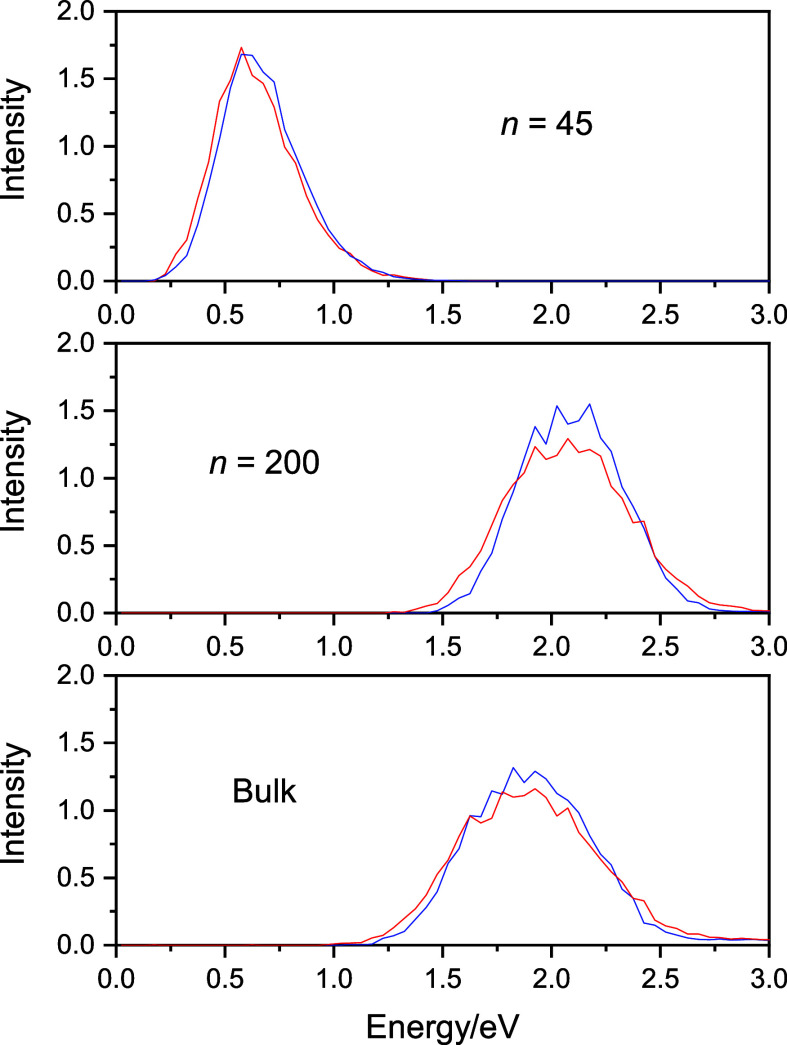
Absorption spectra for the excess electron in water clusters and in the bulk. Classical spectra are blue and quantized are red.

**Figure 2:**
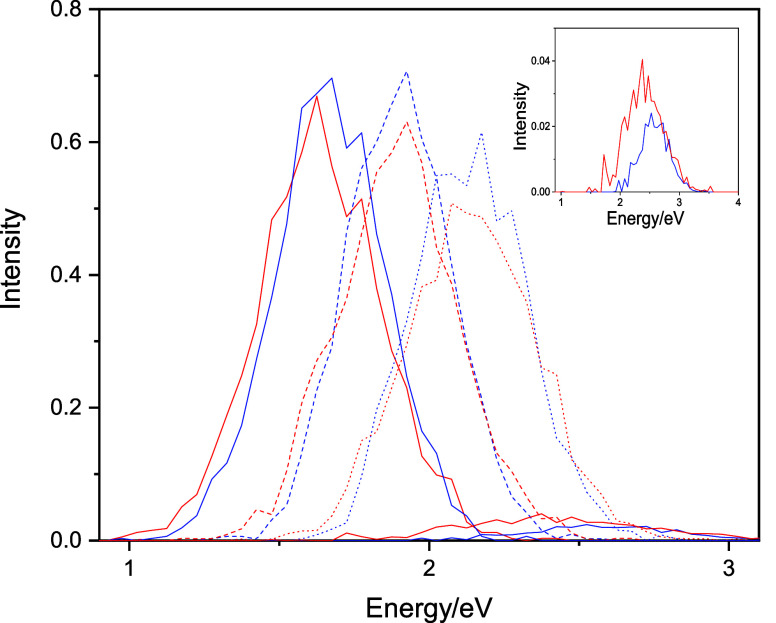
Sub-bands of the first four transitions of the absorption spectrum of the bulk hydrated electron. The figure shows the first three dominant sub-bands and the minor contribution from the fourth transition. The inset shows this small fourth sub-band magnified. Classical spectra are blue and quantized are red.

The computed raw absorption spectra are plotted in Figure 1. Figure 1 clearly shows the apparent characteristics of the excess electron-binding motifs for ensembles of water molecules. Interior-state binding (*n* = 200 cluster and the bulk) appears around the experimentally observed position of the bulk hydrated electron (1.72 eV), while surface-state cluster spectra (*n* = 45) appear at significantly lower energies. Nevertheless, the spectrum of the bulk hydrated electron suffers from significant underestimation of the blue tail of the band. The main reason for this failure is that the computed spectra are dominated only by the first three s–p transitions, as seen in Figure 2. The fourth and higher sub-bands are dwarfed by the sub-bands of the first three transitions.

**Figure 3:**
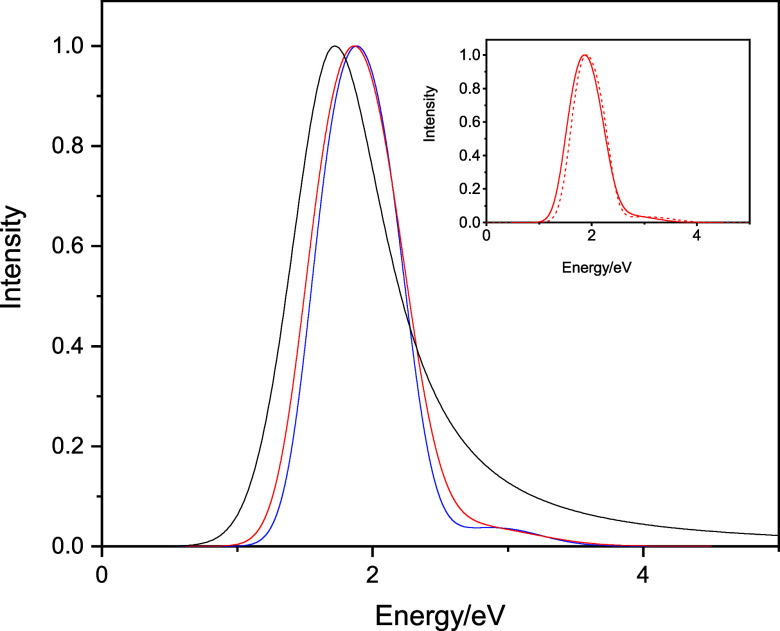
Fitted classical (blue) and the GSTA-quantized (red) spectra for the bulk hydrated electron. The experimental spectrum (black) is also shown for comparative purposes. The spectra are normalized to unity. The inset shows the GSTA-quantized spectrum vs the quantized spectrum based on the autocorrelation quantization scheme.^19^ The absolute peak intensities are 50.9 and 45.3 au for the computed classical and the GSTA-quantized spectra, 60.5 au for the ACF-quantized spectrum,^19^ and 32.8 au for the experiment (corresponding to the maximum molar absorption coefficient, 22,700 dm^3^ mol^–1^ cm^–1^).^86^

More importantly, for our purposes, the difference of the classical and quantized spectra is clear even at the sub-band level. In order to visualize and evaluate the differences between the classical and quantized results, we fitted every band with a Gaussian function and the total spectrum is modeled as the sum of the individually fitted Gaussian functions of the sub-bands. We then compare the attributes of the fitted functions (position and width). The quality of the fits is demonstrated in Figures S1 and S2 of the Supporting Information on the example of the spectrum of the bulk hydrated electron. Figure 3 shows the fitted classical and quantized spectra for the bulk hydrated electron, both normalized to unity at their respective maxima for comparative purposes.

Next, we quantify the NQE using the half width and position of the spectra. The data collected in Table 1 and Figure 1 clearly show that the intensity of the spectra, the position of the maxima, and the half width are all affected by NQEs. The spectra shift moderately to lower energies (by 0.01–0.03 eV) and become broader by 2–15%. The spectra of interior-state electrons are affected significantly more by NQEs (*n* = 200 and bulk), while the changes are more moderate for the surface-state hydrated electron (*n* = 45). It is interesting to point out that the decrease of the intensity (∼20%) and the broadening of the spectra (∼10%) for the bulk hydrated electron compensate each other effectively, resulting only by about 1.5% change in the oscillator strength (the integral) of the spectra. The integral for the classical spectrum is 0.9408 (0.9432 after fitting), while it is 0.9307 (0.9278 after fitting) for the quantized spectrum (Figure S2).

**Table 1:** Maximum Positions and Half Widths of the Fitted Spectra (in eV)^a^

	maximum		half width	
	classical	quantized	classical	quantized
*n* = 45	0.643	0.629 (−14)	0.425	0.432 (+7)
*n* = 200	2.075	2.046 (−29)	0.603	0.703 (+100)
bulk	1.883	1.863 (−20)	0.706	0.773 (+67)

a The shifts are in parentheses (in meV).

To determine whether the observed NQEs in the absorption spectra are statistically significant or not, we calculated the first and the second moments of the absorption spectra.^87^ This way, the confidence intervals can be estimated as well. The zeroth moment of the spectra is its integral (i.e., the oscillator strength, see above). The first moment represents the average frequency, which coincides with the maximum in the symmetric spectrum. The square root of the second moment is the standard deviation of the spectra, which characterizes the broadening of the peak. In the case of the Gaussian line shape, the half width is 2.36 times larger than the standard deviation of the spectra. The details of these calculations are collected in the Supporting Information. The corresponding statistical values are shown in Table 2.

**Table 2:** Means and Standard Deviations of the Absorption Spectra with Confidence Intervals at a Level of 95%

	mean		standard deviation	
	classical	quantized	classical	quantized
QCMD				
*n* = 45	0.674 ± 0.008	0.684 ± 0.009	0.189 (−0.006, +0.006)	0.206 (−0.006, +0.007)
*n* = 200	2.098 ± 0.010	2.084 ± 0.012	0.234 (−0.007, +0.008)	0.284 (−0.009, +0.009)
bulk	1.938 ± 0.015	1.930 ± 0.016	0.332 (−0.010, +0.011)	0.361 (−0.011, +0.012)
TD-DFT				
*n* = 200	1.57 ± 0.08	1.43 ± 0.10	0.25 (−0.05, +0.07)	0.32 (−0.06, +0.09)

Based qualitatively on the confidence intervals, and quantitatively using two-sample one-sided unpaired Student *t* test for comparing the mean positions, and *F*-test for the breadth, we conclude that both the shift of the position of the spectra and the broadening of the cluster spectra upon quantization are likely statistically significant. The shift of the bulk spectrum does not appear to be significant, while the quantum broadening is significant again. Here, we emphasize that NQEs for the broadening reach 10–20%, even for the smaller water cluster anion, as well.

At this point, it is instructive to compare the NQE prediction of the GSTA quantization scheme on the hydrated electron spectrum with the previous procedure using the harmonic quantization scheme on the classical gap–gap and transition dipole moment ACFs.^19^ First, we note at the outset that the position and the breadth of the classical spectrum computed with the ACF approach (1.90 and 0.73 eV)^19^ compared to the present 1.88 and 0.71 eV classical values (Table 1) indicate a slightly blue-shifted and broader spectrum than computed here. Quantization with ACF shifts the spectrum to 1.87 eV,^19^ essentially the same as here (1.86 eV, Table 1), but while the width increases to 0.74 eV in the ACF approach,^19^ more pronounced broadening appears with GSTA to 0.77 eV. Thus, the two quantized spectra plotted in the inset of Figure 3 are similar but closer inspection reveals these fine, nonetheless important differences. The GSTA spectrum predicts a bit red-shifted and noticeably broader spectrum relative to the ACF-based spectrum. On the other hand, the blue tail appears to be more developed in the ACF-based spectrum. This latter effect is due to the fact that in the ACF approach, 12 extra higher lying excited states (*k* > 12) were also incorporated in the calculation by an ad hoc extrapolation procedure.^19^ We also believe that ACF employs several minor approximations in the derivation of its formula (to obtain a tractable mathematical expression) that may influence the outcome. Such approximations are the application of the cumulant expansion to the second order, the neglect of cross-correlation terms, or (perhaps most importantly) the neglect of the energy gap changes upon quantization.^19^ In this respect, GSTA appears to be operating with a smaller set of approximations.

### 3.2 Individual Energy Levels and Gaps

We identify three major components that dominantly contribute to the broadening and shift of the spectra upon quantization. These are (a) the change of the average energy gaps, (b) the broadening of the individual sub-bands, and (c) the change in the splittings between the sub-bands. To examine these components, we analyzed the NQEs on the energy levels and energy gaps within the pseudopotential approach. We find that the individual energy levels lay generally deeper after GSTA quantization than in the classical configurations (see Tables S1 and S2 of the Supporting Information). The decrease of the ground-state energy upon quantization is, however, moderate, around 20 meV which is less than 1% of the ground-state energy (3.08 eV) for the bulk hydrated electron in the classical configurations. The energies of the higher excited states are also higher in the classical case, although the difference becomes smaller at increasingly higher states. The energy gaps can be calculated from the average energy data, and they are collected in Table 3. Also, the energy gap changes upon quantization are shown with confidence intervals in Table S3 of the Supporting Information. Statistical analysis indicates that both the shift of the individual energy levels (Tables S1 and S2) and, more importantly for the present study, the change of the energy gaps (Tables 3 and S3) are statistically significant [with the exception of the energy gaps for the *n* = 45 cluster and the higher (i.e., 0 → 3) transitions].

**Table 3:** Average Energy Gaps with 95% Confidence Intervals for the First Three Dominant Transitions from the Ground State of the Excess Electron^a^

	⟨Δ*E*_01_^*cl*^⟩	⟨Δ*E*_02_^*cl*^⟩	⟨Δ*E*_03_^*cl*^⟩	⟨Δ*E*_01_^*q*^⟩	⟨Δ*E*_02_^*q*^⟩	⟨Δ*E*_03_^*q*^⟩
*n* = 45	0.588	0.665	0.736	0.589 (+1)	0.676 (+11)	0.750 (+14)
	±0.006	±0.007	±0.008	±0.007	±0.008	±0.008
*n* = 200	1.899	2.097	2.293	1.846 (−53)	2.076 (−21)	2.293 (+0)
	±0.007	±0.007	±0.007	±0.008	±0.008	±0.009
bulk	1.671	1.907	2.137	1.633 (−38)	1.893 (−14)	2.136 (−2)
	±0.008	±0.008	±0.008	±0.009	±0.009	±0.010

a The configurations are collected and averaged from the QCMD trajectories (*cl*) and after applying the GSTA procedure (*q*). The shifts are shown in parentheses. Energy gaps are in eV; shifts are in meV.

For the interior-state clusters, the average excitation energy (the average gaps for the three lowest dominant transitions) indicates 20 and 25 meV red shifts for the bulk hydrated electron and for the *n* = 200 cluster anion after quantization. These numbers agree qualitatively well with the shifts observed for the computed spectra (19 and 29 meV in Table 1). For the *n* = 45 surface-state spectrum, the average excitation energies are 0.663 and 0.672 eV, for the classical and the quantized cases, implying a 10 meV blue shift of the quantized spectrum, while spectral calculations predict a 14 meV red shift. For the more nonisotropic surface-state hydrated electron, other factors like the relative intensity of the classical vs quantized sub-bands can play a role in determining the shift of the spectrum. Here, the greater decrease of the intensity of the third sub-band upon quantization pushes the spectrum back to the red-shifted position (see Figure S3 in the Supporting Information).

The nuclear quantum broadening of the spectra (see Table 1) is also the direct consequence of the broadening of the individual sub-bands. Table 4 shows that the broadening varies in the 20–80 meV range depending on the system size, the binding mode of the electron (i.e., surface vs interior states), and the density (temperature) of the systems (200 K in clusters vs 300 K in bulk). Generally, the *n* = 200 cluster anion interior states show 2–3 times greater quantum broadening relative to that of the *n* = 45 cluster anion surface states. This observation points to the possible role of the hydrogen atoms and, in particular, the number of electron–hydrogen interactions (which is about 2–3 times greater in interior states than in surface states) in this phenomenon. Also, lower temperature appears to produce a larger NQE, as we observe in the quantum broadening of the interior states of the 200 K water cluster anions vs 300 K bulk hydrated electron, the former having an approximately 50% greater quantum broadening effect. Furthermore, it is clear that the broadening due to quantization becomes gradually more pronounced for higher transitions.

**Table 4:** Full Widths of the Fitted Sub-bands at Half of the Maximum for the First Three Transitions of the Hydrated Electron^a^

	(0 → 1)^*cl*^	(0 → 2)^*cl*^	(0 → 3)^*cl*^	(0 → 1)^*q*^	(0 → 2)^*q*^	(0 → 3)^*q*^
*n* = 45	0.337	0.352	0.458	0.360 (+23)	0.386 (+33)	0.499 (+41)
*n* = 200	0.358	0.358	0.369	0.423 (+65)	0.426 (+68)	0.451 (+81)
bulk	0.431	0.419	0.456	0.472 (+42)	0.466 (+46)	0.508 (+53)

a The configurations are collected and averaged from the QCMD trajectories (*cl*) and after applying the GSTA procedure (*q*). The quantum broadenings are shown in parentheses. Widths are in eV; broadenings are in meV.

To provide a more quantitative basis for the above observations, we also performed spectral moment analysis on the first three individual sub-bands. The results are listed in Tables S4–S6 in the Supporting Information. The analysis clearly indicates statistically significant quantum broadening of the individual sub-bands and also a shift of the band positions, consistent with the data of Tables 3 and 4 and our previous discussion above.

The third factor that certainly influences the breadth of the optical spectrum is the energy difference, or the splitting, between the sub-bands. Greater splitting implies a broader spectrum. Rossky and Schnitker illustrated that the energy splitting of the bulk hydrated electron, defined as the energy difference between the highest and the lowest energy p states (*E*_3_ – *E*_1_), is strongly correlated to, and therefore, a good measure of the spatial asymmetry of the hydrated electron, of the deviation from spherical symmetry.^21^ The greater the splitting, the greater is the difference between the maximum and minimum values of the moments of inertia tensor of the electron distribution in the principal axis frame. Of the three investigated cases, the analysis is not trivially applicable for the *n* = 45 cluster anion where the electron is localized in an inherently asymmetric (anisotropic) environment. For the *n* = 200 interior-state (cavity-state) cluster anion, we find 394 meV for the classical and 448 meV for the quantized splitting. These values are 466 and 503 meV for the bulk. The 53 ± 3 and 37 ± 3 meV extra splittings (with 95% confidence intervals) are noticeable in comparison with the broadening of the individual sub-bands.

All in all, we think that the combination of three factors is mainly responsible for the major part of the NQEs appearing in the spectrum of the interior state hydrated electrons. We observe that relatively small changes in the individual energy levels lead to larger changes in gaps, broader sub-bands, and greater splittings, subsequently leading to relatively sizable quantum change in the width of the full spectra, up to 20%. We also find it interesting to point out that the confidence intervals for the fluctuations in the individual energy levels are greater than the confidence intervals for the corresponding gaps. This implies that the fluctuations of the individual energy levels take place mostly in a parallel fashion, resulting in smaller deviations of the energy gaps both in the classical and the quantized configurations.

### 3.3 Radius of the Electron and Structural Properties

The structural aspects of the problem are examined next. Important characteristic attributes of the hydrated electron are the radius of gyration of its distribution and the electron (center-of-mass, com)-hydrogen and the electron (com)-oxygen radial distribution functions. The radius of the hydrated electron strongly correlates with the ground-state energy; therefore, one may anticipate changes in radii due to the shifts of the energy levels upon quantization (see above). The average radii in QCMD simulations are 3.96, 2.30, and 2.42 Å for the *n* = 45 cluster, the *n* = 200 cluster, and for the bulk, respectively. The changes in the size of the electron distribution caused by the quantization are, however, almost negligible and are observed to be around 0.01 Å for both the interior and surface states, (−0.013 ± 0.005) Å for *n* = 45, (0.007 ± 0.001) Å for *n* = 200, and (0.008 ± 0.001) Å for the bulk. This is less than 0.5% of the original radii. We note here that while the change of the average size of the electron appears negligible, the electron distribution becomes slightly more asymmetric upon quantization, as we previously pointed out.

**Figure 4:**
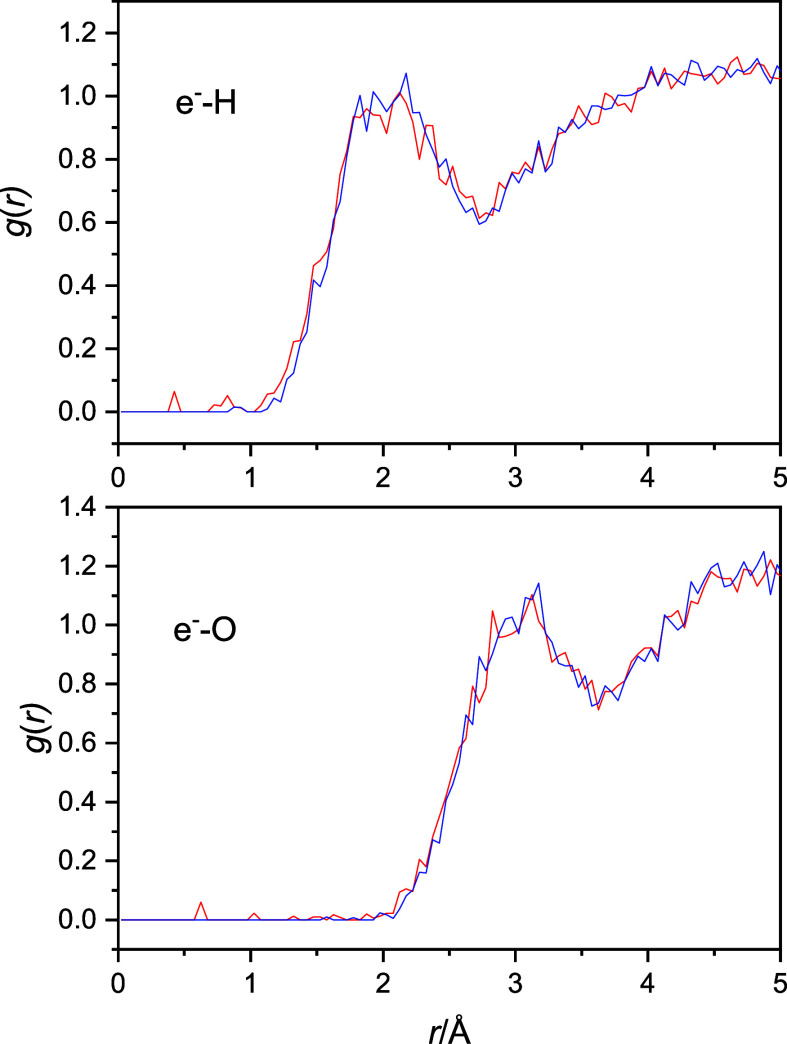
Electron (com)-hydrogen (top) and the electron (com)-oxygen (bottom) radial distribution functions. Classical radial distribution functions are blue and quantized are red.

The electron (com)-hydrogen and electron (com)-oxygen radial distribution functions are shown in Figure 4. Although the functions are very noisy, two quick observations can be made without much risk. (1) The quantized radial distribution functions show some noticeable presence of the atoms in the excluded regions, which is strictly zero in the classical simulations. (2) The quantized functions and their maxima can be discerned to move slightly to shorter distances. These two factors indicate that the OH fluctuations may be greater in the quantized cases, which would also be expected based on some simple consideration of the proton’s anticipated quantum behavior.

**Figure 5:**
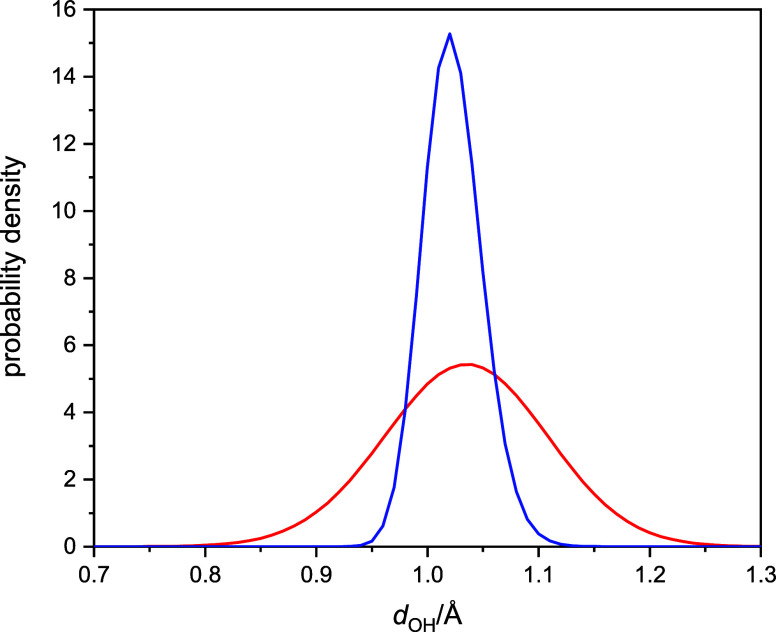
Probability distribution functions of intramolecular OH distances in bulk hydrated electron simulations. Classical distribution is blue and quantized is red.

In fact, this anticipation turns out to be valid, as illustrated in Figure 5. Figure 5 shows the distribution of the intramolecular OH distances computed along the classical and quantized trajectories in the bulk simulations. The significantly broadened quantum distribution indicates large NQE, greater OH fluctuations relative to the classical case, similar to previous results.^53^

### 3.4 Effects of the OH Bond Scaling

**Figure 6:**
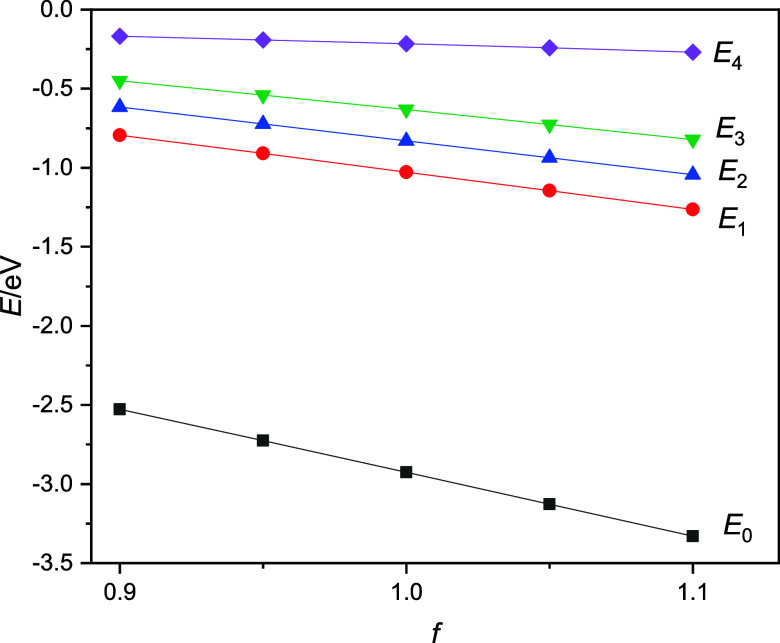
Ground- and excited-state energies of the bulk hydrated electron as a function of the OH distance scaling factors.

Our main hypothesis is that these larger nuclear quantum fluctuations are mainly responsible for the broadening of the spectra of the hydrated electron upon quantization. In the case of larger OH fluctuations, if the OH distance shrinks, then the interaction with the excess electron becomes less favorable, leading to the increase of the ground-state energy of the excess electron and the decrease of the energy gaps for the excitations. If the OH bonds get longer, that results in extra stabilization of the electron, more negative eigenvalues, and increasing energy gaps. We designed a series of calculations to test this hypothesis. First, we took the (classical) configurations of the bulk hydrated electron simulations that we used for the spectral calculations (1996 configurations, see above) and artifactually changed the OH bond lengths while keeping the center of mass of the molecules and the principal axis of inertia fixed. The bond lengths were scaled down or up for all configurations by a series of constant factors, 0.90, 0.95, 1.05, and 1.10, creating four new groups of configurations, two with compressed OH bond lengths and two with elongated OH bonds. We note here that the range of the employed compression and elongation factors is consistent with the quantum distribution function of Figure 5. Next, we calculated the energies and absorption spectra of the hydrated electron within these groups of configurations. For the energies and energy gaps, we observe that the ground-state energy of the excess electron decreases (the electron becomes more stable) and the gaps increase as the bond lengths increase by the OH distance scaling factors from 0.90, 0.95, 1.00 (the classical case), and 1.05 to 1.10. This means that for the configurations with compressed bond lengths, the energy gaps are smaller than in the structures taken from the classical simulation (*f* = 1.00), while in configurations with elongated bonds, the energy gaps increase. These anticipated trends are clearly reflected in Figure 6 with a crudely linear scaling dependency. The relevant data are also collected in Table S7 in the Supporting Information.

**Figure 7:**
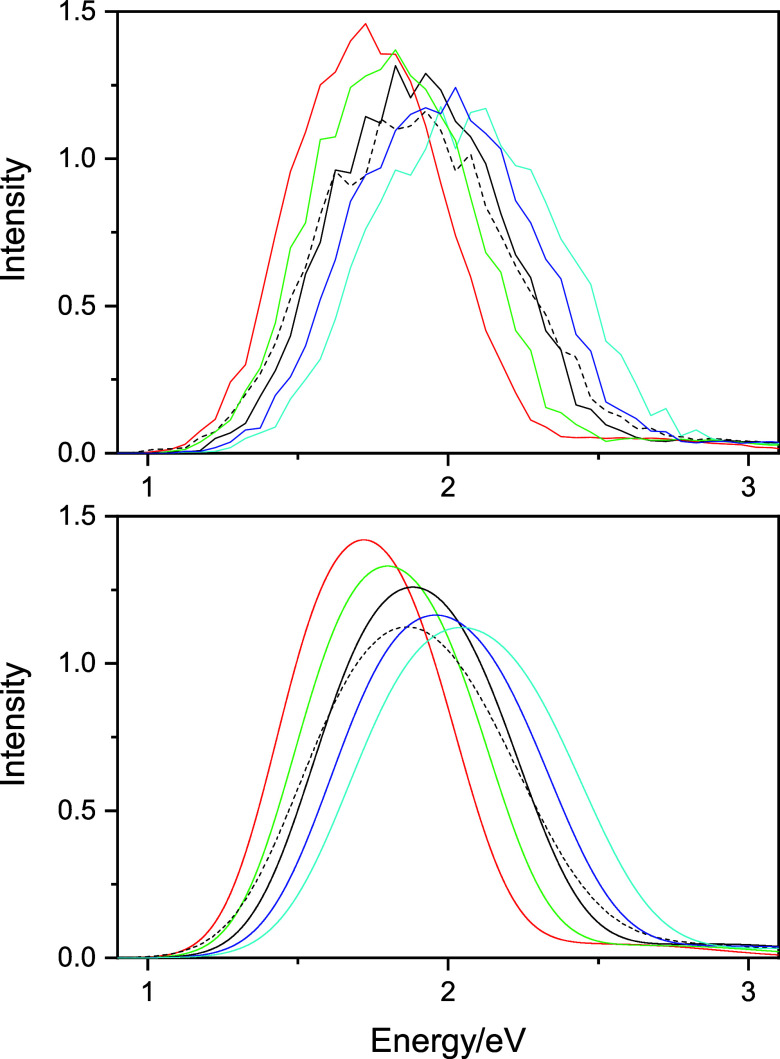
Computed and fitted spectra of the bulk hydrated electron with different OH bond scaling factors. Red: *f* = 0.9, green: *f* = 0.95, black solid: *f* = 1.00 (the classical spectrum), blue: *f* = 1.05, cyan: *f* = 1.1, and black dashed: quantized spectrum.

The direct spectroscopic consequence of the energy and energy gap changes on OH bond scaling is manifested in shifting the corresponding spectra. Assuming that the transition dipoles do not change significantly with alteration of energy gaps, one expects that the absorption spectra for the configurations with compressed bond lengths shift to smaller energies relative to the classical spectrum, while configurations with longer OH distances shift the spectra to greater energies. The computed spectra for the rescaled OH bond configurations underline this anticipation. These spectra are shown in Figure 7. Now, the message from these spectra (and Figure 5) seems clear: the greater fluctuations of the OH lengths in the quantized case introduce both shorter and longer OH bond lengths in the configurations, leading to a broadening of the classically calculated spectrum.

To continue our analysis a bit further, we performed the same set of calculations with changing OH distances for the *n* = 200 cluster anions. The motivation is that the  large interior-state clusters are expected to behave similarly to the bulk and, at the same time, TD-DFT calculations can still be reasonably carried out for these clusters. Not surprisingly, the computed QCMD spectra for the *n* = 200 cluster anions show a pattern of continuous shifting with varying OH bond lengths similar to that for the bulk. These mixed quantum-classical spectra are shown in Figure S4 in the Supporting Information, and the trends can be directly compared to those computed using a higher level of electron structure theory. We present this comparison below.

### 3.5 TD-DFT Calculations

We collected 20 uncorrelated configurations from the *n* = 200 cluster anion QCMD simulations. We then created six sets of these configurations with varying bond lengths. These sets include (1) configurations directly from QCMD simulations (quantum-classical results or *f* = 1.00 scaling factor for the OH bonds), (2) the modified configurations of the first set according to the GSTA procedure, and (3–6) the modified configurations starting from the first set but applying OH length compression or elongation by uniform scaling factors of 0.90, 0.95, 1.05, and 1.10 within the sets. For each set, we computed the TD-DFT spectra considering only the first four electronic transitions, taking 80 transitions into account within one set. For the TD-DFT calculations, we employed the SCAN-D3(BJ) functional and the aug-ma-svp basis set (see the arguments in the Methods section).

Before the detailed analysis, we first compared the QCMD spectrum and the TD-DFT spectrum computed on the first set of configurations that were taken directly from QCMD simulations. The spectra are shown in Figure S5 of the Supporting Information. The TD-DFT spectrum is shifted relative to the QCMD spectrum by about 0.5 eV. Obviously, the two potential energy surfaces are different; therefore, they would sample different parts of the configuration space during their time evolution. The TD-DFT spectrum is computed on QCMD-sampled configurations; therefore, such a shift may not be surprising after all. One possible scenario is that in the classical configurations, the cavity may be a bit too large for the given DFT method. If the QCMD cavities are larger than would be dictated by the DFT potential surface, then the energy gaps decrease (see the compressed OH distance cases and the discussion above). In fact, TD-DFT calculations on the *f* = 1.10 QCMD structures (see below) predict qualitatively the same spectrum as the QCMD (*f* = 1.00) spectrum. Furthermore, shifting the TD-DFT spectrum by 0.5 eV shows that the main features of the spectra are very similar. Based on this observation, we are confident that despite the mismatch of the QCMD and the TD-DFT spectra, the TD-DFT-computed spectra provide additional insights into the origin of the NQEs on the absorption spectrum of the hydrated electron.

For the sake of completeness, we have to mention other sources for the mismatch of the TD-DFT and QCMD spectra, namely, how well the given method, the functional, and the basis set compute the spectrum at given configurations. We anticipate that the use of more diffuse sets would decrease the gaps for higher transitions (where the spatial distribution of the excited state increases) and therefore further increase the present shift. Nevertheless, our guess (which is also based on some limited set of trial calculations) is that the shift of the spectrum is mainly due to the functional.

Before proceeding, an extra observation should also be made about the high-energy tail of the hydrated electron spectrum. It is known, and we have also demonstrated above, that dominantly, the first three transitions contribute to the spectrum in QCMD calculations. This is a well-known limitation of the one-electron QCMD methodology.^15,21,22,59−61^ TD-DFT results, however, indicate significant contribution from the 0 → 4 transition (and supposedly higher transitions, as well) indicating that properly chosen electronic structure methods could and would remedy this problem^65^ (see the bottom part of Figure S5, at above 2.5 eV).

**Figure 8:**
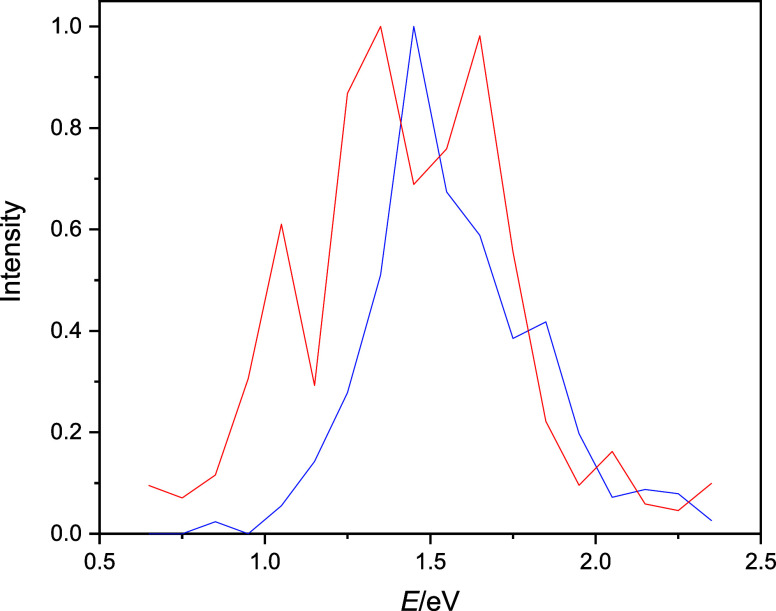
TD-DFT spectra computed on the classical nuclear QCMD configurations (blue curve) and the GSTA-quantized configurations (red curve). The spectra are normalized to unity at their maxima.

Next, we examine the TD-DFT spectra computed on the classical nuclear QCMD configurations and their corresponding GSTA-quantized configurations. Here, to decrease the uncertainty of the conclusions associated with the limited sampling, we added 20 more extra configurations to the original set of 20 configurations and evaluated the spectra on 40 configurations (160 transitions). Figure 8 shows the computed spectra normalized to unity at the maxima. Although significant noise of the data still persists, broadening upon GSTA quantization is evident.

Calculating the mean and the standard deviation of the TD-DFT spectra, we observe that the red shift and the broadening of the peak after the quantization are larger compared to the pseudopotential spectra (see Table 2). Due to the very limited sample size, and the noise of the spectra, it is difficult to assess the statistical significance of these NQEs. Clearly, the red shift of the excitation energies upon quantization is statistically significant (Table S3), and it is directly reflected in the shift of the positions of the individual sub-bands (Tables S4–S6), which is also statistically significant. As an example, for the first energy gap, TD-DFT predicts a 0.21 eV red shift upon quantization (Table S3), while the position of the first sub-band shifts by 0.19 eV (Table S4). The standard deviations of the energy gaps are also strongly related to the breadth of the sub-bands. Here, however, based on the data of Tables 2 and S4–S6, the quantum broadening does not appear to be statistically significant.

**Figure 9:**
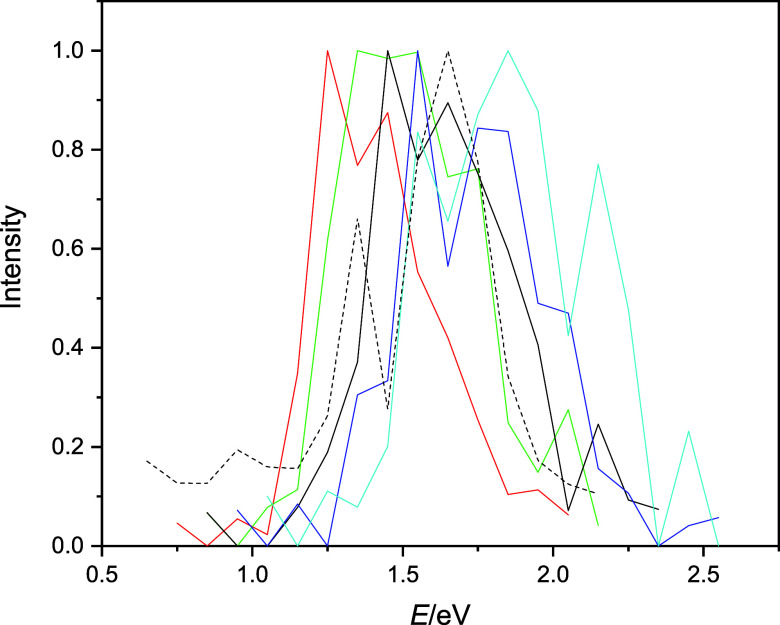
TD-DFT spectra of the *n* = 200 hydrated electron cluster with different OH bond scaling factors applied for the selected QCMD configurations. Red: *f* = 0.9, green: *f* = 0.95, black solid: *f* = 1.00, blue: *f* = 1.05, cyan: *f* = 1.1, and black dashed: quantized configurations. The spectra are normalized to unity at their maxima.

The TD-DFT spectra found on configurations with respect to the compressed/elongated OH bonds result in the same conclusions as we found in the pseudopotential-based calculations above. Compressing the OH bonds in general decreases the energy gap, while increasing the OH bond length increases the gap. The spectra will become shifted accordingly as shown in Figure 9.

In this section, we illustrated that using a higher level of electronic structure methods, all our previous conclusions based on one-electron pseudopotential calculations remain valid. The structural reasons for the broadening spectrum when NQEs are taken into account are associated with greater quantum fluctuations of the OH bonds and, in fact, the larger fluctuation of the hydrogen atoms.

## 4 Conclusions

We examined and analyzed NQEs on the steady-state absorption spectra of the bulk hydrated electron and water cluster anions using the GSTA method. The equilibrium classical nuclear trajectories were generated by one-electron pseudopotential-based QCMD simulations, and these trajectories were transformed (i.e., quantized) by the GSTA method. The absorption spectra computed on the two trajectories clearly demonstrated that the GSTA method indicates sizable NQEs, manifesting mostly in the red shift and the broadening of the spectra. These effects are significantly more pronounced in the case of interior-state excess electrons compared with the surface-bound species. This fact implies the role of those hydrogen atoms in the spectral NQEs that are in direct contact with the excess electron. In fact, the conceptual simplicity of the GSTA method provided a basis on which to comprehend and visualize the microscopic factors that contribute to the NQEs. Here, we point out that the increased quantum fluctuations of the hydrogen atoms directly interacting with the excess electron are predominantly responsible for the broader spectral shape. We also note that in comparison with the previously introduced time correlation function approach, GSTA predicts larger NQEs and also provides a microscopic explanation for the effect.

Previously, the ACF correction represented the most advanced way to estimate NQEs in the absorption spectrum of the hydrated electron. With the application of GSTA, the NQEs in the excitation energies were also considered more accurately. Additionally, NQEs in structural properties were also investigated. With the GSTA approach, it was also possible to use a higher theoretical level than that was used for the simulation. We were able to perform benchmark TD-DFT calculations on selected snapshots, which would not be possible with the ACF quantization scheme. Since the classical and the filtered trajectories are paired data, small NQEs can be detected even at a low number of snapshots. Since the ACF quantization scheme was successfully applied on the lifetime of the excited states, we expect that GSTA can be applied on the investigation of the dynamics of excited states as well.

## Supporting Information Available

Additional details of the simulations and results (PDF)
