# Supplementary material for: 2-in-1 Phase Space Sampling for Calculating the Absorption Spectrum of the Hydrated Electron
Source: J Chem Theory Comput. 2024 May 10;20(10):4265–77. doi: 10.1021/acs.jctc.4c00106 (PMC11137824; doi:10.1021/acs.jctc.4c00106)
Supplement: Supplementary file 1 — ct4c00106_si_001.pdf [file ct4c00106_si_001.pdf]

Supporting Information: 2-in-1 Phase Space Sampling for Calculating the Absorption Spectrum of the Hydrated Electron

László Turi,[*,†] Bence Baranyi,[†] and Ádám Madarász[*,‡]

†*Institute of Chemistry, ELTE, Eötvös Loránd University, Pázmány Péter sétány 1/A, Budapest, H-1117, Hungary*

‡*Research Centre for Natural Sciences, Magyar Tudósok Körútja 2, H-1117 Budapest, Hungary*

E-mail: laszlo.turi@ttk.elte.hu; madarasz.adam@ttk.hu

1 Supplementary Figures

Figure S1.

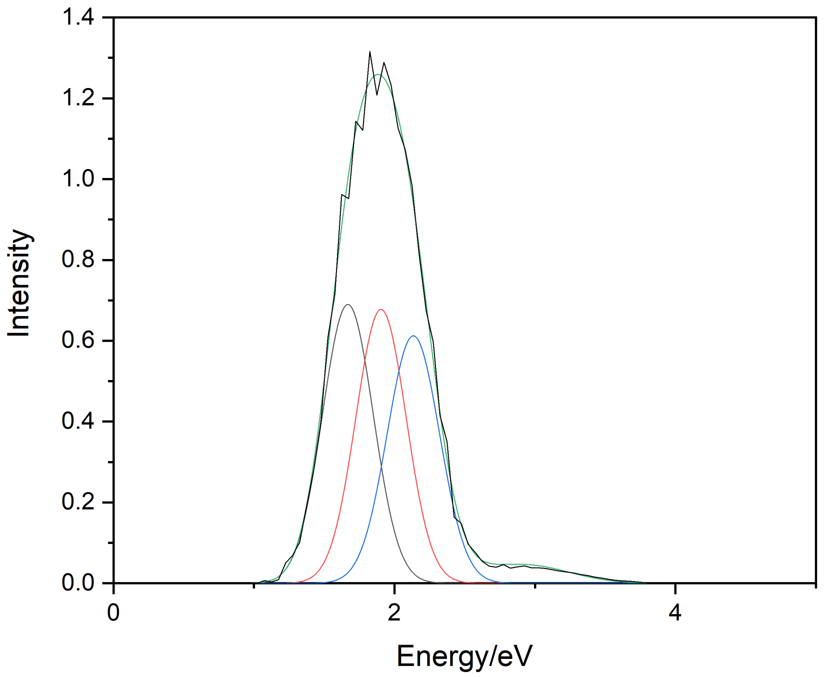

Figure S1. Fitted and computed classical (raw) spectra for the bulk hydrated electron with the first three fitted subbands. The other eight higher transitions are not shown but included in the full spectra.

Figure S2.

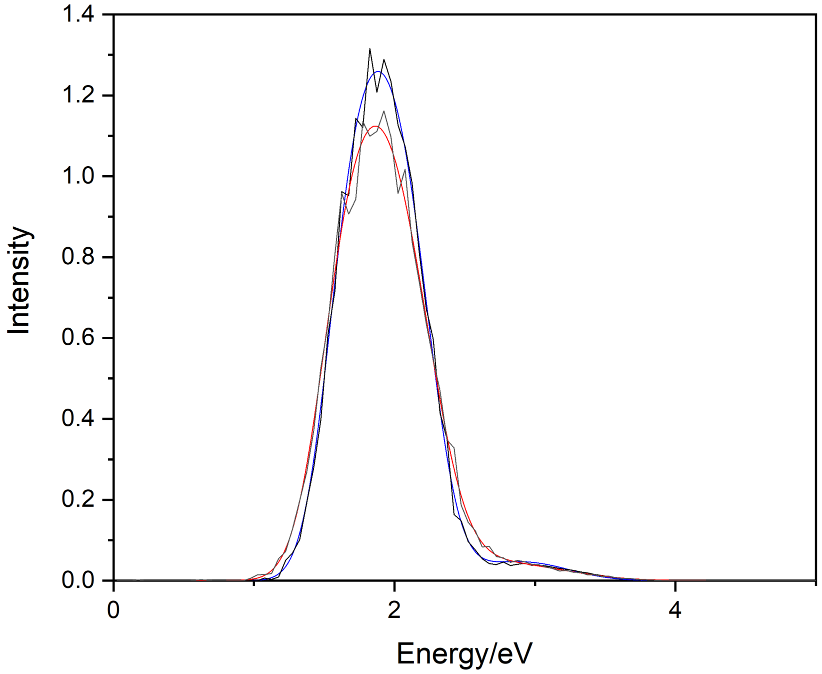

Figure S2. Fitted and computed spectra for the bulk hydrated electron. Raw data are black, the fitted classical spectrum is blue, the fitted quantized spectrum is red. The integral for the classical spectrum is 0.9408 (0.9432 after fitting), while it is 0.9307 (0.9278 after fitting) for the quantized spectrum.

Figure S3.

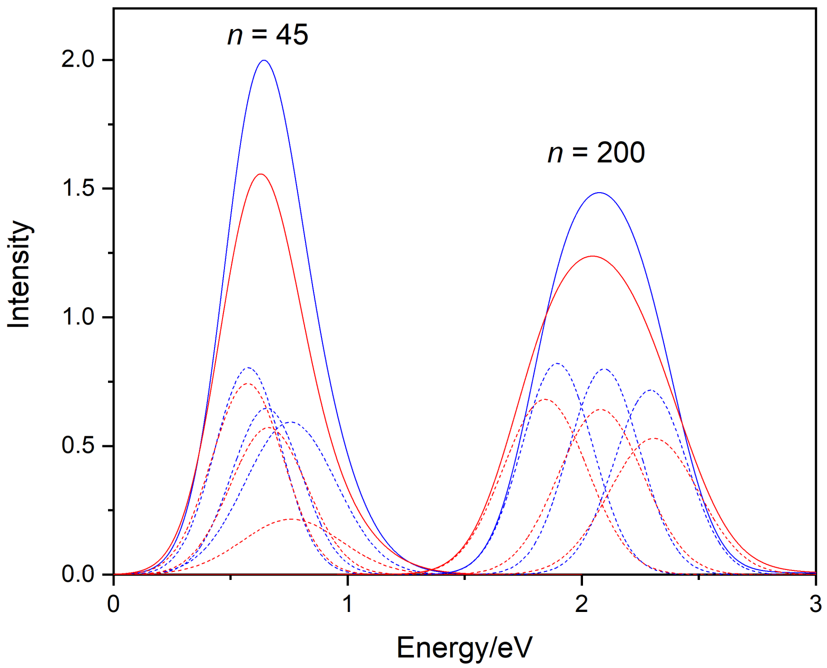

Figure S3. Fitted spectra for the $n = 45$ and the $n = 200$ hydrated electron clusters. The classical spectra are blue, the quantized spectra are red. The first three subbands are also shown by the dashed curves.

Figure S4.

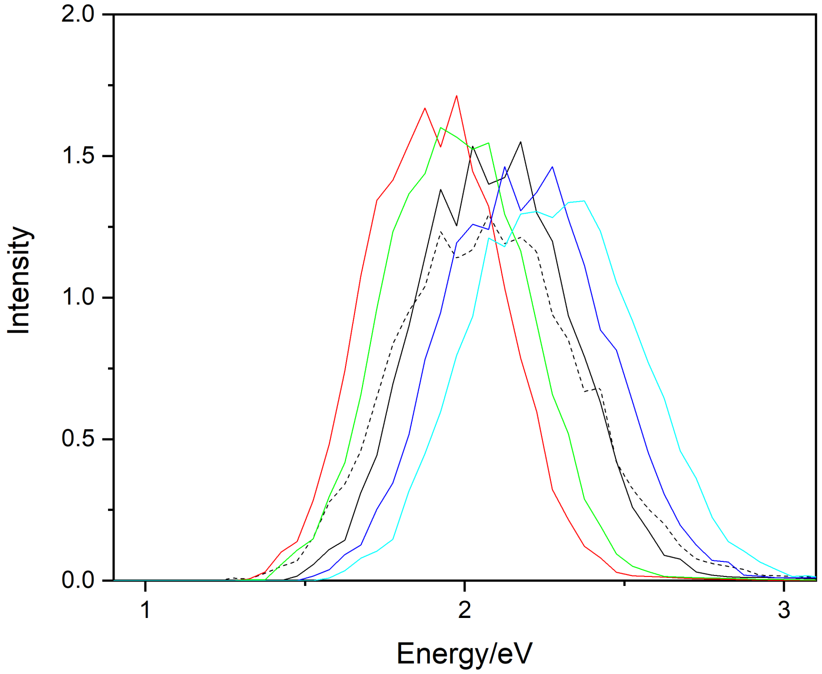

Figure S4. The computed spectra of the $n = 200$ hydrated electron cluster with different OH bond scaling factors. Red: f=0.9, green: f=0.95, black solid: f=1.00 (the classical spectrum), blue: f=1.05, cyan: f=1.1, black dashed: quantized spectrum.

Figure S5.

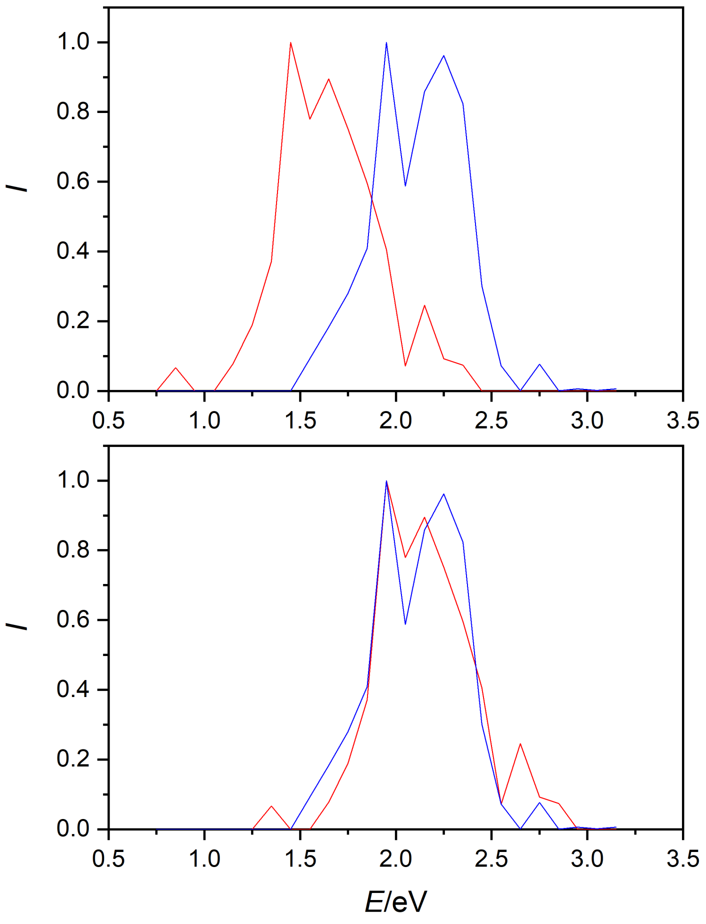

Figure S5. The absorption spectra of the $n = 200$ hydrated electron cluster computed from twenty QCMD configurations using the QCMD method (blue) and TD-DFT calculations (red). The top figure shows the computed and normalized spectra to unity, the bottom figure illustrates the shift of the TD-DFT spectrum by 0.5 eV. The spectra are computed from the first four transitions for each configuration.

2 Error estimation on the nuclear quantum effects

2.1 Nuclear quantum effects on absorption spectra

The absorption spectra is constructed as a sum of the excitation energies X_i weighted with their oscillator strengths f_i. For each system we used $N = 1996$ structures taken from the simulations 100 fs apart from each other. We considered 9 excitation energies in the pseudopotential calculations, thus the absorption spectra were determined from $n = 17964$ data points. The relative weight of the i-th data point can be determined from the oscillator strengths:

$$p_i = \frac{f_i}{\sum\limits_{i=1}^{n} f_i} \tag{1}$$

The mean of an absorption spectrum can be calculated simply as

$$\langle X \rangle = \sum_{i=1}^{n} p_i X_i \tag{2}$$

The standard deviation is

$$s = \sqrt{\sum_{i=1}^{n} \left(X_i - \langle X \rangle\right)^2 p_i} \tag{3}$$

The confidence intervals of the means are calculated as

$$\left[\langle X \rangle - t_{\alpha/2,N-1} \times \frac{s}{\sqrt{N}}, \langle X \rangle + t_{\alpha/2,N-1} \times \frac{s}{\sqrt{N}}\right] \tag{4}$$

where $\alpha = 0.05$ according to the 95% level of significance, t is the Student's distribution.

The confidence interval of the standard deviation is:

$$\left[\sqrt{\frac{N-1}{\chi^2_{\alpha/2,N-1}}}s, \sqrt{\frac{N-1}{\chi^2_{1-\alpha/2,N-1}}}s\right] \tag{5}$$

where χ^2 is the Chi-squared distribution.

2.2 Nuclear quantum effects on paired data

Using GSTA we have paired structures, thus we have paired data X_i^{cl}, X_i^{q}. The mean values calculated as

$$\left\langle X^{\mathrm{cl}}\right\rangle = \frac{\sum_{i=1}^{N} X_i^{\mathrm{cl}}}{N} \tag{6}$$

and

$$\left\langle X^{\mathrm{q}}\right\rangle = \frac{\sum_{i=1}^{N} X_i^{\mathrm{q}}}{N} \tag{7}$$

The difference can be calculated directly on the pairs:

$$\left\langle X^{\mathrm{cl}}\right\rangle - \left\langle X^{\mathrm{q}}\right\rangle = \left\langle X^{\mathrm{cl}} - X^{\mathrm{q}}\right\rangle = \frac{\sum_{i=1}^{N}\left(X_i^{\mathrm{cl}} - X_i^{\mathrm{q}}\right)}{N} \tag{8}$$

The standard deviation is

$$s = \sqrt{\frac{\sum_{i=1}^{N}\left(\left(X_i^{\mathrm{cl}} - X_i^{\mathrm{q}}\right) - \left\langle X^{\mathrm{cl}} - X^{\mathrm{q}}\right\rangle\right)^2}{N}} \tag{9}$$

The confidence interval of the difference can be calculated as above, but the confidence interval of the difference can be smaller than that of X^{cl} or X^{q}.

3 Supplementary Tables

Table S1.

	$\langle E_0^{cl} \rangle$	$\langle E_1^{cl} \rangle$	$\langle E_2^{cl} \rangle$	$\langle E_3^{cl} \rangle$	$\langle E_0^{q} \rangle$	$\langle E_1^{q} \rangle$	$\langle E_2^{q} \rangle$	$\langle E_3^{q} \rangle$
$n = 45$	-0.897	-0.309	-0.231	-0.161	-0.914	-0.325	-0.238	-0.164
	±0.010	±0.005	±0.004	±0.003	±0.012	±0.006	±0.005	±0.003
$n = 200$	-2.926	-1.027	-0.829	-0.633	-2.939	-1.094	-0.864	-0.646
	±0.013	±0.010	±0.010	±0.009	±0.018	±0.015	±0.014	±0.013
Bulk	-3.076	-1.405	-1.169	-0.939	-3.097	-1.464	-1.204	-0.961
	±0.016	±0.013	±0.013	±0.013	±0.021	±0.017	±0.016	±0.016

Table S1. The average individual energy levels with 95% confidence intervals for the ground state and the first three excited states of the excess electron. The configurations are collected and averaged from the QCMD trajectories (cl) and after applying the GSTA procedure (q). Energies are in eV.

Table S2.

	$\langle E_0^{cl} - E_0^q \rangle$	$\langle E_1^{cl} - E_1^q \rangle$	$\langle E_2^{cl} - E_2^q \rangle$	$\langle E_3^{cl} - E_3^q \rangle$
$n = 45$	17.2±2.8	16.2±1.6	6.6±1.4	3.6±0.9
$n = 200$	13.4±7.8	66.5±6.3	34.5±6.1	13.3±5.4
Bulk	20.8±8.2	59.1±6.9	34.4±6.5	22.2±12.5

Table S2. Average decrease of the individual energy levels for the ground state and the first three excited states of the excess electron upon quantization with 95 % confidence intervals. The configurations are collected and averaged from the QCMD trajectories (cl) and after applying the GSTA procedure (q). Energies are in meV.

Table S3.

	$\langle \Delta E^{cl}_{01} - \Delta E^{q}_{01} \rangle$	$\langle \Delta E^{cl}_{02} - \Delta E^{q}_{02} \rangle$	$\langle \Delta E^{cl}_{03} - \Delta E^{q}_{03} \rangle$
QCMD			
$n = 45$	-1.1±1.7	-10.6±1.8	-13.7±2.0
$n = 200$	53.1±3.2	21.1±3.4	0.0±2.0
Bulk	38.3±3.6	13.7±3.6	1.4±4.2
TDDFT			
$n = 200$	209±51	155±46	126±47

Table S3. Average energy gap changes upon quantization with 95 % confidence intervals. The configurations are collected and averaged from the QCMD trajectories (cl) and after applying the GSTA procedure (q). Energy gaps are in meV.

Table S4.

	Mean		Standard deviation	
	classical	quantized	classical	quantized
QCMD				
n=45	0.587±0.006	0.588±0.007	0.144(-0.004,+0.005)	0.157(-0.005,+0.005)
n=200	1.898±0.007	1.848±0.008	0.154(-0.005,+0.005)	0.183(-0.006,+0.006
bulk	1.671±0.008	1.635±0.009	0.182(-0.005,+0.006)	0.206(-0.006,+0.007)
TDDFT				
n=200	1.38±0.07	1.19±0.10	0.22(-0.04,+0.06)	0.30(-0.05,+0.09)

Table S4. Means and standard deviations of the first subband with confidence intervals at level of 95%.

Table S5.

	Mean		Standard deviation	
	classical	quantized	classical	quantized
QCMD				
n=45	0.666±0.007	0.679±0.007	0.152(-0.005,+0.005)	0.167(-0.005,+0.005)
n=200	2.097±0.007	2.082±0.008	0.151(-0.005,+0.005)	0.183(-0.006,+0.006
bulk	1.906±0.008	1.895±0.009	0.177(-0.005,+0.006)	0.205(-0.006,+0.007)
TDDFT				
n=200	1.52±0.07	1.36±0.09	0.22(-0.04,+0.06)	0.28(-0.05,+0.08)

Table S5. Means and standard deviations of the second subband with confidence intervals at level of 95%.

Table S6.

	Mean		Standard deviation	
	classical	quantized	classical	quantized
QCMD				
n=45	0.752±0.008	0.765±0.009	0.188(-0.006,+0.006)	0.206(-0.006,+0.007)
n=200	2.295±0.007	2.312±0.009	0.158(-0.005,+0.005)	0.200(-0.006,+0.006
bulk	2.136±0.008	2.142±0.010	0.184(-0.006,+0.006)	0.217(-0.007,+0.007)
TDDFT				
n=200	1.64±0.07	1.50±0.09	0.21(-0.04,+0.06)	0.27(-0.05,+0.08)

Table S6. Means and standard deviations of the third subband with confidence intervals at level of 95%.

Table S7.

	0.90	0.95	1.00 (classical)	1.05	1.10	quantum
$\langle E_0 \rangle$	-2.528	-2.726	-2.926	-3.127	-3.329	-2.935
$\langle \Delta E_{01} \rangle$	1.734	1.816	1.899	1.982	2.066	1.859
$\langle \Delta E_{02} \rangle$	1.911	2.004	2.197	2.190	2.285	2.082
$\langle \Delta E_{03} \rangle$	2.079	2.187	2.293	2.400	2.507	2.295
$\langle \Delta E_{04} \rangle$	2.360	2.535	2.710	2.885	3.059	2.673

Table S7. Average ground state energies and energy gaps (eV) using QCMD one-electron calculations for various sets of configurations with compressed and elongated OH lengths (see the main text for more details).
